# Quantizing Euclidean Motions via Double-Coset Decomposition

**DOI:** 10.34133/2019/1608396

**Published:** 2019-09-15

**Authors:** Christian Wülker, Sipu Ruan, Gregory S. Chirikjian

**Affiliations:** ^1^Johns Hopkins University, Baltimore, MD, USA; ^2^Department of Mechanical Engineering, National University of Singapore, Singapore

## Abstract

Concepts from mathematical crystallography and group theory are used here to quantize the group of rigid-body motions, resulting in a “motion alphabet” with which robot motion primitives are expressed. From these primitives it is possible to develop a dictionary of physical actions. Equipped with an alphabet of the sort developed here, intelligent actions of robots in the world can be approximated with finite sequences of characters, thereby forming the foundation of a language in which robot motion is articulated. In particular, we use the discrete handedness-preserving symmetries of macromolecular crystals (known in mathematical crystallography as Sohncke space groups) to form a coarse discretization of the space SE(3) of rigid-body motions. This discretization is made finer by subdividing using the concept of double-coset decomposition. More specifically, a very efficient, equivolumetric quantization of spatial motion can be defined using the group-theoretic concept of a double-coset decomposition of the form Γ\SE(3)/Δ, where Γ is a Sohncke space group and Δ is a finite group of rotational symmetries such as those of the icosahedron. The resulting discrete alphabet is based on a very uniform sampling of SE(3) and is a tool for describing the continuous trajectories of robots and humans. An efficient coarse-to-fine search algorithm is presented to round off any motion sampled from the continuous group of motions to the nearest element of our alphabet. It is shown that our alphabet and this efficient rounding algorithm can be used as a geometric data structure to accelerate the performance of other sampling schemes designed for desirable dispersion or discrepancy properties. Moreover, the general “signals to symbols” problem in artificial intelligence is cast in this framework for robots moving continuously in the world.

## 1. Introduction

The aim of this paper is to develop a “motion alphabet” with which robot motion primitives are expressed. From these primitives one can develop a dictionary of physical actions. Two main themes from the theory of Lie groups are used to construct this alphabet (or quantization) and to efficiently solve the “signals to symbols” problem in this context: (1) the decomposition of a Lie group into cosets, double cosets, and corresponding fundamental domains; (2) the possibility of constructing such fundamental domains as Voronoi or Voronoi-like cells.

Equipped with an alphabet of the sort developed in this paper and the associated algorithms for efficiently rounding off continuous motions to nearby discrete representatives, intelligent actions of robots in the world can be approximated with finite sequences of characters, thereby forming the foundation of a language in which robot motion is articulated.

At the macroscopic scale, the world can be thought of as continuous. Coarse descriptions of this continuous world are used by intelligent systems (*e.g.*, humans and computers) to classify objects, actions, and scenarios. The classical “signals to symbols” problem in artificial intelligence (AI) seeks to bin everything in the continuous world into countable classes characterized by strings of discrete symbols, such as the letters in an alphabet. In a sense, this is the inverse problem of what the genetic code does, since the finite alphabet {A, C, G, T} encodes the morphology and metabolism of every living creature that moves in the continuous world and processes information with an analog brain.

Discrete alphabets (including the Roman alphabet, the radicals that form characters in Asian languages, sign language, etc.) form the basis for all human languages [1]. All discrete characters can be reduced to a binary code,* e.g.*, ASCII or Morse code in the case of western letters. The efficiency of an alphabet (or a code) depends on how much information can be conveyed with a given number of symbols, and how difficult it is to convey those symbols. For example, since the letter “e” is the most often used symbol in the English language, it is represented by a single “·” in Morse code. Perhaps the simplest and most widely occurring motion of a robot is the “nil motion”, or identity element of the motion group, describing the “home” pose (position and orientation), which coincidentally can be denoted simply as “*e*”. All other elements of our motion alphabet will be written as a product of the form *γδ* where *γ* ∈ Γ, a crystallographic space group, and *δ* ∈ Δ, a finite group of rotational symmetries.

As an example of how discrete symbols classify the world, consider how each of the following sentences describes a class of situations in which there is continuous freedom which becomes more restricted as the number of discrete symbols increases:

1. The cat is on the table.
2. The black cat is sitting on the table.
3. The black cat is sitting on the table and looking at you.

In (1), the cat could be of any color and lying down, sitting, or standing in an infinite variety of postures. The color and posture of the cat get somewhat restricted as the sentences get longer, but we still do not know how the cat is sitting, what its tail is doing, how big it is, its weight, the color of its eyes, etc. Indeed, the sentence would need to be quite long to hone in on what is actually going on, hence the old saying “one picture is worth a thousand words.”

The basic problem that must be reconciled by intelligent robots is to approximate, or round off, continuous objects and actions within a discrete descriptive framework and then truncate the discrete description at some finite level given limitations on computational and sensing resources. This is true in both machine-learning (ML) frameworks such as deep learning and classical AI. A specific kind of rounding off (of Euclidean motions) is the main subject of this paper, which is about making precise the round-off statement

(1) $$\begin{array}{c} discrete\;\;description=\left[continuous\;\;description\right] \end{array}$$

in the context of motions of objects and intelligent agents in the world.

A real-world problem that can be addressed using this discrete description of motions is robot motion planning, which is one of the fundamental topics in the field of intelligent robotics. It has a wide range of applications such as autonomous vehicles [2–4], mechanical parts assembly [5, 6], space on-orbit manipulations [7], and protein folding [8]. In general, motion planning seeks to answer the query: “how to plan a path that guides the robot from a given start pose to an end pose subject to some geometric or dynamical constraints”. And a popular way that has been applied for decades is to build a “roadmap” [9], which is basically a graph structure consisting of valid vertices and edges. A large number of efficient algorithms have been proposed such as visibility graph [10], Voronoi graph [11], cell decomposition [12], probabilistic roadmap [13], and their variants. For a given environment and constraints (obstacles, nonholonomic constraints, etc.), a roadmap provides information with qualified vertices and connectivity among nearby vertices. And once a query is submitted, graph searching algorithms give a valid optimal sequence of motions from the roadmap. Although a roadmap method is able to answer multiple searching queries, when the environment is changing, those vertices or edges information needs to be updated. To some extent, quantizing a continuous motion of the robot from a predefined motion alphabet is closely related to the roadmap concept. However, the advantage lies in the storage and representation of the motion sequences—once a rich library of alphabets is built a priori, representing different motions in different environment subject to different constraints is just a matter of combinations and ordering of the alphabet indices.

The major contributions of this paper are listed as follows:

(1) A “signals to symbols” framework for Euclidean motions based on double-coset decomposition is proposed and its properties are analyzed.

(2) Concrete formulations of the motion alphabet are constructed through crystallographic symmetry.

(3) Fast decoding algorithms via coarse-to-fine double-coset decomposition are proposed and numerically verified.

(4) Comparisons with existing methods for discretizing the Euclidean group and decoding random motions are performed.

(5) A fast hybrid search method that incorporates some existing sampling methods for rotation group with good dispersion or discrepancy properties has been proposed and verified.

The remainder of this paper is structured as follows: In Section 2, a review of the immense literature on machine intelligence as it applies to intelligent robot action in the world is provided. This is a rapidly changing field and impossible to capture in full detail, but some classical highlights are covered.  Section 3 reviews some relevant aspects of abstract group theory. This is made concrete in Section 4 which focuses on the group of rigid-body motions.  Section 5 reviews crystallographic symmetry, which is a source of discrete symbols from which a motion alphabet is constructed. In Section 6, we develop a motion alphabet by “dividing up” the group SE(3) of rigid-body motions via fine double-coset decomposition based on a crystallographic Sohncke space group and the subgroup of rotational symmetries of the icosahedron.  Section 7 presents other choices for motion alphabets and solves the decoding problem efficiently by introducing a coarse-to-fine search scheme. In Section 8, comparisons with existing methods for sampling rotations and Euclidean motions are performed, and it is shown how our alphabet can be used as a geometric data structure to enhance the speed of these other motion-approximation methods.

## 2. Literature Review

This section reviews two largely disjoint areas of the literature: (1) the interface between group theory and machine learning and AI; (2) sampling methods and measures of their quality.

### 2.1. Lie Groups in Machine Learning and AI

The recognition of human (and humanoid-robot) actions has been studied from many different perspectives including [14–19]. Probabilistic graphical models [20], generative models [21], and recently “SE3-nets” [22] have been used to describe motion uncertainty in the context of learning. Works on vision and reasoning use concepts of quotient operations to mod out irrelevant information [23–25].

Group-theoretic methods (and abstract algebra more generally) can be found sprinkled throughout the AI literature [26–32]. Although group theory has long history [33–35], it is still a useful and popular tool for solving problems related to motions [36–40].

Of particular importance in the current context is the relationship between artificial intelligence and machine learning. AI arose as a branch of cybernetics, focusing on artificial aspects of reasoning and cognition, thereby leading to a redefinition of cybernetics to focus on information and control. Machine learning (and particularly deep neural networks) led by Hinton, LeCun, Bengio, and others can be viewed as an alternative to classical AI [41–45]. Recently, geometric and algebraic methods are being explored in some forms of machine learning [46–48].

The goal of this paper is to develop an alphabet of basic motions from which discrete words that capture the essence of a continuous motion/action are constructed. Throughout the literature, discretization of motions has attracted significant interest, where the Euclidean group is one of the popular ones [49]. In particular, the uniform sampling of rotations, either random or deterministic, has a wide range of applications [50–56]. This is also one of the applications that this work will address. The discretization of the continuous motions will generate part of a dictionary. This dictionary will, in the future, serve as the knowledge base for an expert system that will enable the robot to function at first use “right out of the box.”

### 2.2. Measurements of Sample Quality: Discrepancy, Dispersion, Consistency, and Uniformity

Sampling rotations in an efficient way play important roles in several fields including computer graphics [50, 57], protein crystallography [58], molecular physics [59], materials science [60], and robot motion planning [53, 55].

Several measures of the quality of a finite sampled set of rotations have been proposed in the literature. One is* discrepancy* as defined and used in [53, 61, 62]:

(2) $$\begin{array}{c} D\left(P,R\right)≔\underset{R\in R}{sup}\left|\frac{\left|P\cap R\right|}{\left|P\right|}-\frac{Vol\left(R\right)}{Vol\left(SO\left(3\right)\right)}\right|, \end{array}$$

where *ℛ* is a collection of measurable subsets of SO(3), *P* is a finite set of sample points in SO(3), and |*S*| is the number of elements in the finite set *S*. This concept was updated recently to include products of motion groups [63].

Natural measures of distance between rotations, or between rigid-body motions, can be computed in a variety of different ways, as reviewed in detail in [64].

Given any such metric, *ρ* : SO(3) × SO(3) → *ℝ*_≥0_, the* dispersion* of the points can be computed as

(3) $$\begin{array}{c} D\left(S,\rho \right)≔\underset{R\in SO\left(3\right)}{max}\left[\underset{R_{S}\in S}{min}\;\rho \left(R,R_{S}\right)\right], \end{array}$$

where *S* is a set of the sampled elements in SO(3).

In addition to discrepancy and dispersion, many other measures of sample quality can be defined. For example, a sampling can be called* consistent* if the distribution of the round-off error for any random rotation is concentrated. Therefore, we define the consistency of a set of samples on SO(3) as

(4) $$\begin{array}{c} C\left(S,\rho \right)≔\sigma_{R\in S}\left\{m\left(R\right)\right\}, \end{array}$$

where

(5) $$\begin{array}{c} m\left(R\right)≔\underset{R_{S}\in S-\left\{R\right\}}{min}\;\rho \left(R,R_{S}\right) \end{array}$$

and *σ* is the standard deviation of the set of distances between each sample point and its nearest neighbor. Here a low value of *ℭ*(*S*, *ρ*) indicates high consistency. For example, if *ℭ*(*S*, *ρ*) is zero, then every point in the set has nearest neighbors of the same distance. Moreover, if each point in the set has many neighbors that achieve the minimal value of distance, then the set *S* has a high level of* uniformity*, which can be quantified as follows. For each *R* ∈ *S* compute the subset *A*_*R*_ ⊂ *S* as

(6) $$\begin{array}{c} A_{R}≔\left\{Q\in S:\rho \left(R,Q\right)=m\left(R\right)\right\}. \end{array}$$

Then the cardinality of this set measures the number of equally close nearest neighbors to *R*, and uniformity weights this by the spread of this number:

(7) $$\begin{array}{c} U\left(S,\rho \right)≔\frac{\min_{R\in S}\;\left|A_{R}\right|}{1+\max_{R\in S}\;\left|A_{R}\right|-\min_{R\in S}\;\left|A_{R}\right|}, \end{array}$$

where a high value indicates high uniformity. The numerator reflects the number of nearest neighbors for the worst sample, and the denominator reflects the spread (with 1 included since the difference between max and min can be zero).

For example, an integer lattice in 3D Euclidean space has a high level of uniformity with regard to the Euclidean metric, with each point having six nearest neighbors, each with the same minimized value of distance, and so *ℭ*(*S*, *ρ*) = 0 and *𝔘*(*S*, *ρ*) = 6. A spherical close-packing can have an even higher value of *𝔘*(*S*, *ρ*).

## 3. Some Relevant Aspects of Group Theory

The alphabets constructed in this paper consist of carefully chosen elements of the group of rigid-body motions, drawn from fundamental domains of double-coset spaces. Since this terminology might not be familiar to some readers with an interest in the topic, the relevant concepts from group theory are reviewed here. The concept of a group itself is assumed to be known.

### 3.1. Definitions and Properties

Let a group *G* and a subgroup *H* < *G* be given. For an element *g* ∈ *G*, the corresponding* left coset* is defined as

(8) $$\begin{array}{c} gH≔\left\{gh:h\in H\right\}. \end{array}$$

Analogously, the respective* right coset* is defined as

(9) $$\begin{array}{c} Hg≔\left\{hg:h\in H\right\}. \end{array}$$

A subgroup *N* < *G* is called a* normal* subgroup of *G* if it is conjugated to itself; *i*.*e*., *gNg*^−1^ = *N* for all *g* ∈ *G*. If *N* is both a normal and a proper subgroup of *G*, then it is denoted as *N*◁*G*.

A group can be divided into cosets (left or right), all with the same number of elements, and the set of all these cosets is called a* coset space*. In general, the left-coset space

(10) $$\begin{array}{c} G/H≔\left\{gH:g\in G\right\} \end{array}$$

and the right-coset space

(11) $$\begin{array}{c} H\backslash G≔\left\{Hg:g\in G\right\} \end{array}$$

are different from each other, except when *H* is normal in G (*H*◁*G*), in which case *gH* = *Hg* for all *g* ∈ *G*. In this special case *G*/*H* = *H*\*G*≕*H*/*G* is a* factor* (or* quotient*)* group*.

Given two subgroups *H*, *K* < *G*, it is also possible to define* double cosets*

(12) $$\begin{array}{c} HgK≔\left\{hgk:h\in H,\;\;k\in K\right\},\;g\in G, \end{array}$$

and the corresponding* double-coset space*,

(13) $$\begin{array}{c} H\backslash G/K≔\left\{HgK:g\in G\right\}. \end{array}$$

Given a left-coset decomposition with respect to a subgroup *H*, it is possible to define a (nonunique)* fundamental domain*

(14) $$\begin{array}{c} F_{G/H}\subset G \end{array}$$

consisting of exactly one element per left coset. Since a group can be partitioned into disjoint cosets, it is

(15) $$\begin{array}{c} G=\underset{g\in F_{G/H}}{⋃}gH \end{array}$$

and

(16) $$\begin{array}{c} G=\underset{h\in H}{⋃}F_{G/H}h \end{array}$$

(and analogously in the right-coset case [65]). It is important to note that we are generally dealing with unions of disjoint sets in this paper. When *H*◁*G*, such fundamental domain is a group with respect to the original group operation modulo *H*, and this group is isomorphic to *G*/*H*.

Given two subgroups *H*, *K* < *G*, the corresponding double-coset decomposition is

(17) $$\begin{array}{c} G=\underset{g\in F_{H\backslash G/K}}{⋃}HgK \end{array}$$

and we further have that

(18) $$\begin{array}{c} G=\underset{h\in H}{⋃}\;\underset{k\in K}{⋃}hF_{H\backslash G/K}k, \end{array}$$

where *F*_*H*\*G*/*K*_ is a fundamental domain for the double-coset space *H*\*G*/*K* consisting of exactly one element per double coset. When *G* is a* Lie group* (*i.e.*, when *G* has the structure of a differentiable manifold, and when further the group operation and inversion are compatible with this smooth structure) and *H*, *K* are* discrete* subgroups, then such fundamental domains *F*_*G*/*H*_ and *F*_*H*\*G*/*K*_ will have the same dimensionality as *G*, but smaller volume.

When *G* is a Lie group, one way to construct fundamental domains is as Voronoi-like cells: Since *G* is a smooth manifold, a proper distance function (metric) *ρ* exists, and we can define

(19) $$\begin{array}{c} F_{G/H}^{\circ }≔\left\{g\in G:\rho \left(e,g\right)<\rho \left(e,gh\right)\text{ for all}\;\;h\in H\backslash \left\{e\right\}\right\},\\ F_{H\backslash G}^{\circ }≔\left\{g\in G:\rho \left(e,g\right)<\rho \left(e,hg\right)\text{ for all}\;\;h\in H\backslash \left\{e\right\}\right\}, \end{array}$$

where *e* is the identity element of *G*, and when *H*∩*K* = {*e*},

(20) $$\begin{array}{c} F_{H\backslash G/K}^{\circ }≔\left\{g\in G:\rho \left(e,g\right)<\rho \left(e,hgk\right)\text{ for all}\;\;\left(h,k\right)\in H\times K\backslash \left\{\left(e,e\right)\right\}\right\}. \end{array}$$

Above we have defined the* interior* of fundamental domains. The union of the corresponding shifts of these open sets will not in general completely exhaust G (cf. (16) and (18)). However, a set of measure zero will at most be missing, which is not relevant for our practical purposes.

A* unimodular* Lie group *G* is one for which there exists an integration measure, *μ*, or equivalently a volume element *dg*, such that for every integrable function *f* : *G* → *ℝ*,

(21) $$\begin{array}{c} \mu \left(f\right)≔\int_{G}f\left(g\right)dg=\int_{G}f\left(g_{0}g\right)dg=\int_{G}f\left(gg_{0}\right)dg=\int_{G}f\left(g^{-1}\right)dg. \end{array}$$

In the case of a compact Lie group such as *G* = *SO*(3), *μ*(1) = *Vol*(*G*).

In terms of *ZXZ* Euler angles (*α*, *β*, *γ*), it is well known that the volume element for SO(3) is [64]

(22) $$\begin{array}{c} dg=sin\;\beta \;d\alpha \;d\beta \;d\gamma . \end{array}$$

Consequently, sampling uniformly in Euler angles leads to clumping of samples around *β* = 0 and *π*, and under sampling near *β* = *π*/2.

Some authors therefore sample *β* nonuniformly, by making a change of coordinates as *β* = cos^−1^(*x*). Then $sin\;\beta =\sqrt{1-x^{2}}$, and $\left|d\beta /dx\right|=1/\sqrt{1-x^{2}}$. This results in equivolumetric portioning of *SO*(3) in the coordinates (*α*, *x*, *γ*) with volume element *dg* = *dα*  *dx*  *dγ*.

However, equipartitioning into units of equal volume is not the same as equipartitioning into units of equal shape. One attempt to partition based on shape arose nearly 50 years ago in the context of protein crystallography, where Lattman [58] realized that the metric tensor for SO(3) as expressed in Euler angles (*α*, *β*, *γ*) could be diagonalized by changing to (*α*′, *β*′, *γ*′)≔(*α* + *γ*, *β*, *α* − *γ*). This diagonalization does not change the volume element, which remains *dg* = sin *β*′  *dα*′  *dβ*′  *dγ*′.

Group theory has been a cornerstone in all areas of the physical sciences including crystallography, classical mechanics, quantum mechanics, chemistry, and particle physics. Moreover, in classical works on finite automata theory, attempts were made to incorporate the theory of finite groups [26–28]. Many roboticists and computer vision researchers know about the special Euclidean group SE(3), which is a Lie group that describes rigid-body motions. This will be discussed later, after first reviewing the group of pure rotations.

### 3.2. A Concrete Example: The Rotation Group

In the following, the abstract definitions are illustrated with a concrete example. The set of 3 × 3 rotation matrices is called the* special orthogonal group* and is denoted as SO(3). That is,

(23) $$\begin{array}{c} SO\left(3\right)≔\left\{R\in R^{3\times 3}:R^{T}R=I\;\;and\;\;det\;R=1\right\}, \end{array}$$

where *𝕀* is the identity matrix. Here the group operation is simply matrix multiplication. It is not difficult to show that *𝕀* is the group identity, given any two *R*_1_, *R*_2_ ∈ SO(3) that the matrix product *R*_1_*R*_2_ ∈ SO(3), and that *R*^−1^ = *R*^T^ is the inverse of *R* ∈ SO(3).

Explicitly for SO(3), elements of the associated Lie algebra *𝔰𝔬*(3), which correspond to infinitesimal rotations, are* skew-symmetric matrices*

(24) $$\begin{array}{c} X=\left[\begin{array}{c} \begin{array}{ccc} 0 & -x_{3} & x_{2}\\ x_{3} & 0 & -x_{1}\\ -x_{2} & x_{1} & 0 \end{array} \end{array}\right], \end{array}$$

and the* matrix exponential* (or* exponential*)* map* gives

(25) $$\begin{array}{c} R\left(x\right)=exp\left(X\right)=I+\frac{sin{\left\|x\right\|}_{2}}{{\left\|x\right\|}_{2}}X+\frac{1-cos{\left\|x\right\|}_{2}}{{\left\|x\right\|}_{2}^{2}}X^{2}, \end{array}$$

where **x** = (*x*_1_, *x*_2_, *x*_3_)^T^≕*X*^∨^ is the dual vector corresponding to *X*. The opposite operation gives $\hat{x}≔X$. The parameters *x*_1_, *x*_2_, and *x*_3_ can be thought of as Cartesian coordinates in the Lie algebra *𝔰𝔬*(3), and when these coordinates are restricted to the range ‖**x**‖_2_ ≤ *π*, they can be used to parameterize all of SO(3) through the exponential map. When ‖**x**‖_2_ = *π* the point is at the boundary. In such a case **x** and −**x** describe the same rotation, and so one model for SO(3) is that of a solid ball of radius *π* in Euclidean space, with antipodal points identified as being equivalent, or “glued.”

The inverse map for each is the* matrix logarithm*. This degenerates when the rotation angle *θ*≔‖**x**‖_2_ is *π*. By restricting the discussion to the case when *θ* < *π*, the logarithm is uniquely defined on a subset of SO(3) depleted by the set of measure zero defined by *θ* = *π*. This depletion will have no effect on our formulation. Indeed, we can define the metric

(26) $$\begin{array}{c} \rho \left(R_{1},R_{2}\right)≔{\left\|{log}^{\vee }\left(R_{1}^{T}R_{2}\right)\right\|}_{2} \end{array}$$

when *R*_1_^T^*R*_2_ is not a rotation by *π*, and otherwise *ρ*(*R*_1_, *R*_2_)≔*π*.

It is interesting to note that the above distance function *ρ* for SO(3) is* bi-invariant; i.e.*,

(27) $$\begin{array}{c} \rho \left(R_{1},R_{2}\right)=\rho \left(RR_{1},RR_{2}\right)=\rho \left(R_{1}R,R_{2}R\right),\;R\in SO\left(3\right). \end{array}$$

Using this particular metric *ρ* it is possible to construct* Voronoi cells* (in the classical sense) in SO(3) for fundamental domains *F*_*H*\SO(3)_ and *F*_*H*\SO(3)/*K*_, because then (19) and (20) become

(28) $$\begin{array}{c} F_{SO\left(3\right)/H}^{\circ }=F_{H\backslash SO\left(3\right)}^{\circ }=\left\{R\in SO\left(3\right):\rho \left(R,I\right)<\rho \left(R,h\right)\text{ for all}\;\;h\in H\backslash \left\{I\right\}\right\} \end{array}$$

and

(29) $$\begin{array}{c} F_{H\backslash SO\left(3\right)/K}^{\circ }=\left\{R\in SO\left(3\right):\rho \left(R,I\right)<\rho \left(R,hk\right)\text{ for all}\;\;\left(h,k\right)\in H\times K\backslash \left\{\left(I,I\right)\right\}\right\}, \end{array}$$

respectively.

**Figure 1:**
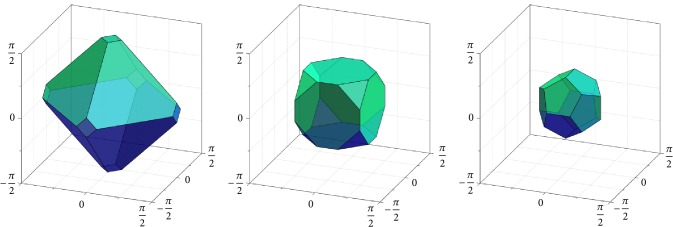
Fundamental domain *F*_*H*\SO(3)_ with *H* as the group of (from left to right) tetrahedral, octahedral, and icosahedral symmetries, constructed as Voronoi cells, viewed in exponential coordinates.

Of particular interest to us are the cases where *H* is one of the finite groups of rotational symmetries of the Platonic solids. This is shown in Figure 1 (see also [54]). In this figure the fundamental domains *F*_*H*\SO(3)_ are depicted in exponential coordinates in *𝔰𝔬*(3) (identified with a ball of radius *π*, as explained above). Note that this is a conceptual plot, since actually the edges and faces of these Voronoi cells are slightly bent. The number |*H*| of elements in *H* is 12 for the group of tetrahedral, 24 for the group of octahedral, and 60 for the group of icosahedral rotational symmetries. By the left (or right) action of *H* on the respective fundamental domain, it is possible to (almost completely) cover SO(3); cf. (16).

The coset spaces resulting from quotienting the rotation group by discrete subgroups have been studied in the pure mathematics literature under the names “spherical space forms” [66] and Poincaré homology 3-spheres [67]. The geometric and topological properties of these manifolds are related to how the opposing faces of our tiles can be glued together.

**Figure 2:**
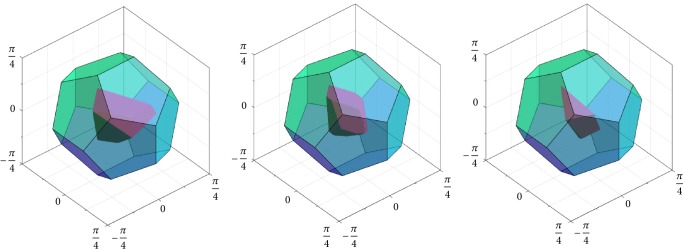
Center Voronoi cell *F*_*H*\SO(3)_ in coset space (emerald region) with *H* as the group of icosahedral symmetries, and center Voronoi cell *F*_*H*\SO(3)/*K*_ in double-coset space (ruby region) with *H* as the group of icosahedral symmetries for all cases and *K* as a conjugated group of (from left to right) tetrahedral, octahedral, and icosahedral symmetries, respectively.

**Figure 3:**
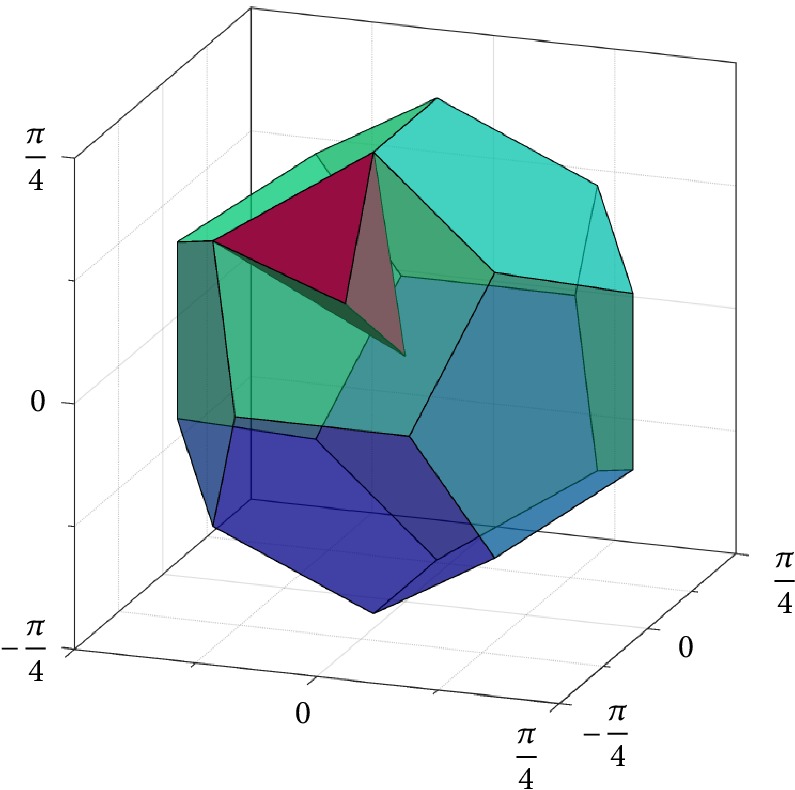
Dodecahedral cell *F*_*H*\SO(3)_ (emerald region) and tetrahedral wedge *F*_*H*\SO(3)/*H*_ (ruby region), with *H* as the group of icosahedral symmetry. The dodecahedral cell can be decomposed into 60 identical tetrahedral wedges, with five packed on each pentagonal face.

If *H* is the group of rotational symmetry operations of the icosahedron, then |*H*| = 60 and *F*_*H*\SO(3)_ can be viewed as a dodecahedral cell centered at the origin of the Lie algebra *𝔰𝔬*(3) (see Figure 1, right). If we choose the second subgroup *K* to be a conjugated group of the tetrahedral, octahedral, or icosahedral symmetries (*i.e.*, *K*≔*gPg*^−1^, where *P* is the group of the rotational symmetries of the respective Platonic solid and *g* ∈ SO(3) is chosen such that *H*∩*K* = {*𝕀*}), then the Voronoi cell *F*_*H*\SO(3)/*K*_ takes a shape as exemplarily shown in Figure 2. On the other hand, if we choose *K*≔*H*, then *F*_*H*\SO(3)/*H*_ cannot be constructed as a Voronoi cell, but it can be chosen as an irregular tetrahedron (the ruby region in Figure 3), yielding a subdivision of the dodecahedral cell *F*_*H*\SO(3)_ by conjugation of the tetrahedron with the elements in *H*. Note that such conjugation has the effect of rotation in *𝔰𝔬*(3) since log^∨^(*gRg*^−1^) = *g* log^∨^*R*. Similarly, if *K* < *H*, then a |*K*|-fold division of *F*_*H*\SO(3)_ can be computed to represent *F*_*H*\SO(3)/*K*_, and *F*_*H*\SO(3)_ can be reconstructed from these pieces, again by conjugation.

When choosing *H* to be icosahedral and *K* = *H* or *K* = *gHg*^−1^, this means that we can divide SO(3) into 3600 pieces of equal size. The 3600 respective barycentric or Voronoi centers of these cells can be taken as a discretization of SO(3), and any of these centers can be written in a unique way as *R*_*ij*_ = *h*_*i*_*k*_*j*_ where (*h*_*i*_, *k*_*j*_) ∈ *H* × *K* with *i*, *j* ∈ {1,2,…, 60}. This means that any one of the 3600 points *R*_*ij*_ corresponds to a two-letter word (*h*_*i*_, *k*_*j*_).

A natural question to ask is then, for *R* ∈ SO(3), how do we find the closest word (*h*_*i*_, *k*_*j*_) to approximate it? This decoding or “signals to symbols” problem is addressed in Section 7.1.

## 4. Rigid-Body Motions as a Group Used for Framing Robots

Given any rigid object or multi-rigid-body actor (such as a human, robot, self-driving car, or smart house), the behavior and use of this item involve the time evolution of its conformation and its overall position and orientation, or “pose”. A common descriptive framework for both the internal (conformational) degrees of freedom and their relative pose (configuration) is to attach reference frames on each rigid component.

Let *g* = (*R*, **t**) denote a rigid-body motion relative to a reference frame fixed in space, where *R* ∈ SO(3) is a rotation matrix and **t** ∈ *ℝ*^3^ is a translation vector. The set of all such motions forms a six-dimensional Lie group, the* special Euclidean group S*E(3). This is a group because the composition operation

(30) $$\begin{array}{c} g_{1}g_{2}=\left(R_{1},t_{1}\right)\left(R_{2},t_{2}\right)≔\left(R_{1}R_{2},R_{1}t_{2}+t_{1}\right) \end{array}$$

satisfies the properties of closure and associativity, the identity exists and is simply *e* = (*𝕀*, 0) (with zero translation), and the inverse of *g* is *g*^−1^ = (*R*^T^, −*R*^T^**t**). Note that SE(3) is not commutative (Abelian). The group operation is the same as multiplying homogeneous transformation matrices;* i.e.*,

(31) $$\begin{array}{c} H\left(g_{1}g_{2}\right)=H\left(g_{1}\right)H\left(g_{2}\right),\;where\;\;H\left(g\right)≔\left[\begin{array}{c} \begin{array}{cc} R & t\\ 0^{T} & 1 \end{array} \end{array}\right]. \end{array}$$

In the case of* planar* motions, we deal with the special Euclidean group SE(2), where *R* ∈ SO(2) is parameterized by a single angle *θ*, and **t** ∈ *ℝ*^2^ has components (*x*, *y*), totalling three degrees of freedom. SO(2) is the group of rotations in *ℝ*^2^, defined analogously to (23). In general, SE(*d*) is an example of an* outer* (or* external*)* semidirect product* which combines the two groups (*ℝ*^*d*^, +) and SO(*d*) into the new group

(32) $$\begin{array}{c} SE\left(d\right)≔SO\left(d\right)⋉R^{d}. \end{array}$$

The underlying set of this group is the Cartesian product SO(*d*) × *ℝ*^*d*^, but the symbol *⋉* reflects the fact that the group operation is not simply (*R*_1_, **t**_1_)(*R*_2_, **t**_2_) = (*R*_1_*R*_2_, **t**_1_ + **t**_2_), which is also a group (called the* direct product*), but does not reflect the way that rigid-body motions work.

The next section reviews the handedness-preserving (Sohncke) crystallographic space groups, which are discrete subgroups of SE(3) and which form an important component of the motion alphabets developed in this paper.

## 5. Crystallographic Groups

A* crystallographic group* is a discrete (and also cocompact) subgroup of the* Euclidean group *E(*d*)≔O(*d*)⋉*ℝ*^*d*^, where O(*d*) is the* orthogonal group* consisting of all orthogonal real-valued *d* × *d* matrices (defined as in (23) for *d* = 3, but also allowing det *R* = −1 there). In addition to rotations, the group O(*d*) also contains* reflections* and* rotoreflections* (*improper rotations*). If *d* = 3, a crystallographic group is commonly called a* space group*, for *d* = 2, it is referred to as a* wallpaper group*. The literature on mathematical crystallography is immense and spans many decades. Some notable introductions include [68–71]. The relationship between torsion-free crystallographic groups (*i.e.*, Bieberbach groups; see below) and flat manifolds has been studied extensively [72–76].

Elements of a crystallographic group Γ can be expressed as pairs

(33) $$\begin{array}{c} \gamma =\left(R_{\gamma },t_{\gamma }+v\left(R_{\gamma }\right)\right), \end{array}$$

where *R*_*γ*_ ∈ *ℙ* (a discrete* point group*,* i.e.*, a subgroup of O(*d*)), **t**_*γ*_ ∈ *𝕃* (a lattice in *ℝ*^*d*^), and **v** : *ℙ* → *ℝ*^*d*^. In particular, **v** is the translational part of a glide-reflection or screw-displacement lattice motion. In general **v** will satisfy the* cocycle identities*

(34) $$\begin{array}{c} v\left(I\right)=0,\\ v\left(R_{\gamma_{1}}R_{\gamma_{2}}\right)=\left(R_{\gamma_{1}}v\left(R_{\gamma_{2}}\right)+v\left(R_{\gamma_{1}}\right)\right)mod\;T, \end{array}$$

where *T*≔{*𝕀*}⋉*𝕃* is the subgroup of pure (or* primitive*) translations in Γ, which is always normal (*T*◁Γ). The “mod *T*” removes components in the sum that are in *T*, in analogy with, for example, (1 + 5)mod 4 = 2 in modulo-4 arithmetic.

If an element *γ* ∈ Γ\{*e*} is of* finite order* (*i.e.*, if there exists an *n* ∈ *ℕ* such that *γ*^*n*^ = *e*), it is called a* torsion element*. The group Γ is called* torsion-free* (or a* Bieberbach group*) if it is free of torsion elements. This is equivalent to the property that no element *γ* ∈ Γ other than the identity *e* has a fixed point (*i.e.*, a point **p** ∈ *ℝ*^*d*^ with *γ ***p** = **p**). If **v** ≡ 0 in (33), then Γ can be written as the semidirect product Γ = *ℙ*⋉*𝕃* and the group is called* symmorphic*. Of the 230 possible types of space groups, 73 can be decomposed in this way. These are the* symmorphic space groups*. Bieberbach groups are not symmorphic.

In the theory of crystallographic groups, a well-known isomorphism is due to the above-mentioned fact that the translation subgroup *T* of Γ is normal:

(35) $$\begin{array}{c} \frac{\Gamma }{T}≅P. \end{array}$$

Moreover, it can be shown that *ℙ* is not only discrete, but must be* finite*. In the case *d* = 3 the point group *ℙ* will belong to one of 32 discrete* crystallographic point groups* that constitute the so-called* crystal classes*.

In the context of space groups, we can distinguish between Bieberbach (*i.e.*, torsion-free) groups as one extreme, and groups which contain only the identity and torsion elements (rotations, reflections, and improper rotations) as the other extreme, with all other space groups lying somewhat “in between.” For a symmorphic space group Γ, a Bieberbach subgroup Γ_B_ < Γ with minimum index in Γ is the subgroup *T* of primitive translations; for many nonsymmorphic space groups, on the other hand, there is a Bieberbach subgroup Γ_B_ with index [Γ : Γ_B_]<[Γ : *T*] allowing for a decomposition of Γ as a group product [77, p. 719]

(36) $$\begin{array}{c} \Gamma =\Gamma_{B}S≔\left\{\gamma_{B}s:\gamma_{B}\in \Gamma_{B},s\in S\right\}, \end{array}$$

where *S* < Γ is a proper subgroup of *ℙ*⋉{0} < E(3), and thus Γ_B_∩*S* = {*e*}. If Γ_B_◁Γ, then (36) is an* inner* (or* internal*) semidirect product (Γ = Γ_B_⋊*S*). In this case, the decomposition *γ* = *γ*_B_*s* is unique for each *γ* ∈ Γ. Another useful property is that each of the 65* Sohncke space groups* (*i.e.*, handedness-preserving space groups Γ < SE(3)) can be written as a product

(37) $$\begin{array}{c} \Gamma =\Gamma_{B}\Gamma_{S}≔\left\{\gamma_{B}\gamma_{S}:\gamma_{B}\in \Gamma_{B},\;\;\gamma_{S}\in \Gamma_{S}\right\}, \end{array}$$

where Γ_B_ and Γ_S_ are, respectively, Bieberbach and symmorphic subgroups (see [77, Thm. 3]). If Γ_B_∩Γ_S_ = {*e*} and Γ_B_ or Γ_S_ is normal, then (37) is an inner semidirect product, and for every *γ* ∈ Γ there exist unique *γ*_B_ ∈ Γ_B_ and *γ*_S_ ∈ Γ_S_ such that *γ* = *γ*_B_*γ*_S_. If both Γ_B_ and Γ_S_ are normal, then Γ is decomposed into the inner direct product Γ = Γ_B_ × Γ_S_.

## 6. A Motion Alphabet Based on a Fine Double-Coset Decomposition

The essence of language is communicating information about the continuous world using a finite number of symbols, characters, or patterns. In the same spirit, in classical AI, a fundamental goal is to convert perceptual information from the continuous world to strings of symbols drawn from a finite alphabet, so that the machinery of finite-state automata reasoning can be applied. Every action of a robot in the physical world can be described as a sequence of paths in SE(3). Such paths are continuous mathematical trajectories which can be viewed as “signals”.

A fundamental problem in AI and sensory perception is the so-called “signals to symbols” problem [78–80], in which observations in the continuous world are converted to coarsified representations (*i.e.*, symbols), on which AI systems can execute logical reasoning algorithms. A natural way to discretize the space of paths is to first discretize time, thereby reducing the infinite dimensions of the path space to a finite number of dimensions, and then to replace each sampled pose on the path with a rounded-off version in a discrete set (the alphabet of maneuvers). Because SE(3) does not have dense subgroups, a clever discretization needs to be constructed.

In the three-dimensional case, one of the finest space groups is Γ = P432 which has a total of 24 rotational elements corresponding to the rotational symmetry operations of a cube (cf. [69, p. 634f]). Therefore, if we want to quantize rigid-body motions with a resolution that is reasonable for real-world tasks, something finer than rounding off to the nearest element of Γ is necessary.

Building on ideas introduced in the context of protein-packing models in X-ray crystallography [55, 65, 77, 81, 82], we can augment Γ by an auxiliary discrete rotation group. For example, let Δ denote the group of rotational symmetries of the icosahedron (as a discrete subgroup of SE(3)). Δ has 60 elements, and there is no lattice of discrete translations that corresponds to it. In particular, Γ, Δ < SE(3) and Γ∩Δ = {*e*}, the identity element. This means that the double-coset space Γ\SE(3)/Δ is a compact Riemannian manifold. It is possible to define a compact fundamental domain *F*_Γ\SE(3)/Δ_ ⊂ SE(3) using (20). Restricting the quotient map from SE(3) to Γ\SE(3)/Δ to the fundamental domain *F*_Γ\SE(3)/Δ_ then gives a bijective (*i.e.*, one-to-one) mapping from *F*_Γ\SE(3)/Δ_ to Γ\SE(3)/Δ. Moreover, the action of Γ (from the left) and Δ (from the right) gives a way to tile SE(3) with disjoint shifts of *F*_Γ\SE(3)/Δ_, because (cf. (18))

(38) $$\begin{array}{c} SE\left(3\right)=\underset{\gamma \in \Gamma }{⋃}\;\underset{\delta \in \Delta }{⋃}\gamma F_{\Gamma \backslash SE\left(3\right)/\Delta }\delta . \end{array}$$

Intensive study of the fundamental domains *F*_Γ\SE(3)_ and *F*_Γ\SE(3)/Δ_ has been conducted (*ibid.*).

**Figure 4:**
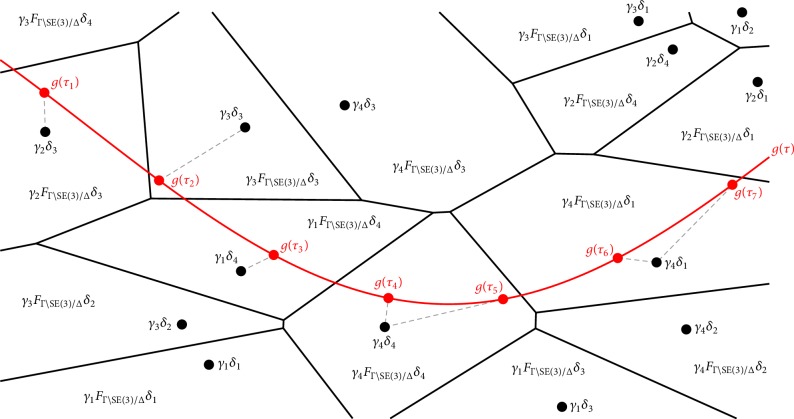
Discretizing a continuous motion trajectory *g* at times *τ*_1_,…, *τ*_7_ using the alphabet Γ × Δ (conceptual plot). After discretization the continuous motion can be expressed as the sentence (*γ*_2_, *δ*_3_), (*γ*_3_, *δ*_3_), (*γ*_1_, *δ*_4_), (*γ*_4_, *δ*_4_), (*γ*_4_, *δ*_4_), (*γ*_4_, *δ*_1_), (*γ*_4_, *δ*_1_).

When the fundamental domain *F*_Γ\SE(3)/Δ_ is constructed using (20), it has the identity element *e* at its center, and so the tiling in (38) has the effect of sampling each center point by moving from *e* to *γδ* where *γ* ∈ Γ and *δ* ∈ Δ. In other words, the product Γ × Δ < SE(3)^2^ can be used as a quantized version of SE(3). The number of rotational elements will be 24 × 60 = 1440 which is sufficiently fine to capture the essence of any frame along a trajectory during a robot task. The alphabet defined by Γ × Δ is infinite, but by limiting the extent of translations to be contained in a bounded region, it becomes finite. This means that continuous trajectories can be translated into a finite string of alphabet characters (Figure 4). This opens up the possibility of mapping these quantized trajectories into words expressed in a natural language.

An important advantage of the quantization scheme (38) is that the shifted fundamental domains *γF*_Γ\SE(3)/Δ_*δ* will all have the same volume;* i.e.*,

(39) $$\begin{array}{c} \mu \left(\gamma F_{\Gamma \backslash SE\left(3\right)/\Delta }\delta \right)=\mu \left(F_{\Gamma \backslash SE\left(3\right)/\Delta }\right)\;\text{for all}\;\;\left(\gamma ,\delta \right)\in \Gamma \times \Delta , \end{array}$$

where *μ* is the (left- and right-invariant) Haar measure on the (unimodular) Lie group SE(3). This means that the centers *γδ* of these shifted fundamental domains used for quantization at the same time also allow for a very uniform sampling of the group SE(3).

## 7. More Alphabets and Coarse-to-Fine Decoding Algorithms

As explained above, after wisely designing a pair of discrete subgroups Γ, Δ < SE(3) with Γ∩Δ = {*e*}, we can construct a fundamental domain as in (20) and decompose SE(3) as in (38). There are many other ways to choose *F*_Γ\SE(3)_ and *F*_Γ\SE(3)/Δ_, as explained in [65]. A particularly simple choice is the Cartesian product

(40) $$\begin{array}{c} F_{\Gamma \backslash SE\left(3\right)/\Delta }≔F_{P\backslash SO\left(3\right)/\Delta }\times F_{L\backslash R^{3}}, \end{array}$$

where *ℙ*≅Γ/*T* is the point group of Γ and *𝕃* the lattice of primitive translations. Alternatively, given decomposition (36), another natural choice is

(41) $$\begin{array}{c} F_{\Gamma \backslash SE\left(3\right)/\Delta }≔F_{S\backslash SO\left(3\right)/\Delta }\times F_{\Gamma_{B}\backslash R^{3}}. \end{array}$$

Here *S*\SO(3)/Δ and Γ_*B*_\*ℝ*^3^ are not (double-)coset spaces—because *S*≮SO(3) and Γ_B_≮*ℝ*^3^—but are* orbit spaces* consisting, respectively, of* orbits SR*Δ≔{*sRδ* : *s* ∈ *S*, *δ* ∈ Δ} and Γ_*B*_**x**≔{*γ*_B_**x** : *γ*_B_ ∈ Γ_B_} (*R* ∈ SO(3), **x** ∈ *ℝ*^3^). The fundamental domains *F*_*S*\SO(3)/Δ_ and *F*_Γ_*B*_\*ℝ*^3^_ above can be constructed by choosing exactly one point per orbit, which is consistent with the definitions in Section 3. Different fundamental domains *F*_Γ\SE(3)/Δ_ such as those above can be used to express different quantizations via (38).

### 7.1. The Purely Rotational Case

The choice for *F*_Γ\SE(3)/Δ_ in (40) allows us to bootstrap off of the fundamental domains for double-coset spaces for SO(3) discussed earlier. In fact, we can go even further and describe an SE(3) motion trajectory *g*(*τ*) = (*R*(*τ*), **t**(*τ*)) in SO(3) × *ℝ*^3^ (as a direct product rather than a semidirect product). This is not merely to make things easier—viewing pose change trajectories in this way has some advantages, as described in [40], where the direct product SO(3) × *ℝ*^3^ is called the* pose change group* and is denoted as PCG(3). Therefore, below we describe in some detail how the “signals to symbols” problem can be solved efficiently in the purely rotational case.

Since (40) is a set rather than a group, we can view it as a subset of either SE(3) or PCG(3). Either way, the general decoding problem reduces to this: Given *H*, *K* < SO(3), and *R* ∈ SO(3), how can we efficiently find the unique pair (*h*_*i*_, *k*_*j*_) ∈ *H* × *K* such that

(42) $$\begin{array}{c} R=h_{i}Qk_{j} \end{array}$$

with *Q* ∈ *F*_*H*\SO(3)/*K*_? In particular, if the Voronoi choice is made for *F*_*H*\SO(3)/*K*_, solving (42) allows for simply rounding off *R* to *h*_*i*_*k*_*j*_, as indicated in Section 3.2.

The question then becomes how to do this. With the crystallographic constraint, in SE(3) it is possible to define *H* such that |*H*| = 24 (octahedral symmetry) and |*K*| = 60 (icosahedral symmetry), leading to 24 × 60 = 1440 combinations. In PCG(3), on the other hand, subgroups need not be restricted to the crystallographic constraint, and we can have more rotational elements (see below). The question is, is there a better way to test for *h*_*i*_ and *k*_*j*_ than two nested for loops over *i* and *j* resulting in a large number of evaluations to find where *ρ*(*R*, *h*_*i*_*k*_*j*_) is minimized, which is equivalent to solving (42) when the Voronoi choice is made for *F*_*H*\SO(3)/*K*_?

The answer is positive, and we shall now present a technique to achieve this. Consider the double-coset space *H*\SO(3)/*K*, where *H* is the group of rotational symmetries of the icosahedron and *K* = *gHg*^T^ is a conjugated group, with *g* being chosen so that *H*∩*K* = {*𝕀*}. It is thus |*H*\SO(3)/*K*| = |*H*| × |*K*| = 60^2^ = 3600. We can construct a fundamental domain *F*_*H*\SO(3)_ for the coset space *H*\SO(3) as a dodecahedral Voronoi cell (cf. (28) and Figure 1, right). Due to the Voronoi property, we can find the shifted tile *h*_*i*_*F*_*H*\SO(3)_ containing the rotation *R* of interest by computing the distance *ρ*(*R*, *h*_*i*_) of *R* to the 60 tile centers *h*_*i*_ ∈ *H*. We then know that *h*_*i*_^T^*R* lies in the identity-centered tile *F*_*H*\SO(3)_. We can construct the fundamental domain *F*_*H*\SO(3)/*K*_ for the double-coset space as a Voronoi cell, too (cf. (29) and Figure 2, right). Since the shifts *h*_*i*_*F*_*H*\SO(3)/*K*_*k*_*j*_ of this identity-centered fundamental domain will cover SO(3), they will also cover *F*_*H*\SO(3)_. However, the number of required shifts will be much smaller than 3600. Indeed, we found that approximately 180 shifted fundamental domains *h*_*i*_*F*_*H*\SO(3)/*K*_*k*_*j*_ are sufficient when the conjugation element *g* is empirically chosen, for example, to minimize the extent

(43) $$\begin{array}{c} \underset{R\in F_{H\backslash SO\left(3\right)/K}}{sup}\rho \left(R,I\right) \end{array}$$

of *F*_*H*\SO(3)/*K*_. We chose *g* in this particular way so as to minimize the round-off error in the quantization scheme. We can now quickly find the shifted fundamental domain *h*_*i*′_*F*_*H*\SO(3)/*K*_*k*_*j*_ containing the above *h*_*i*_^T^*R* by exploiting the Voronoi property of these shifted domains. We then have that *R* ∈ (*h*_*i*_*h*_*i*′_)*F*_*H*\SO(3)/*K*_*k*_*j*_, and so the decomposition (42) is found. Instead of the 3600 distances on SO(3), in the above technique, we have to compute only about 60 + 180 = 240 distances, resulting in a potential speedup of approximately 3600/240 = 15. We further exploited the fact that *ρ*(*R*_1_, *R*_2_) = arccos((1/2)[tr(*R*_1_^T^*R*_2_) − 1]), the trace wherein can be computed efficiently as

(44) $$\begin{array}{c} tr\left(R_{1}^{T}R_{2}\right)=\overset{3}{\underset{i=1}{\sum }}\;\overset{3}{\underset{j=1}{\sum }}{\left[R_{1}\right]}_{ij}{\left[R_{2}\right]}_{ij}, \end{array}$$

as well as the fact that arccos *x* < arccos *y* if and only if *x* > *y*.

An even more efficient decoding algorithm can be obtained when considering the double-coset space *H*\SO(3)/*H*, where *H* is the group of rotational icosahedral symmetry. Here again we can use the dodecahedral Voronoi fundamental domain *F*_*H*\SO(3)_ for the coset space *H*\SO(3) and find the shifted tile *h*_*i*_*F*_*H*\SO(3)_ containing the rotation *R* easily as described above. As a fundamental domain *F*_*H*\SO(3)/*H*_ for the double-coset space, we can choose the tetrahedral wedge shown in Figure 3. We can find the conjugated wedge *h*_*j*_*F*_*H*\SO(3)/*H*_*h*_*j*_^T^ containing the “pulled-back” rotation *h*_*i*_^T^*R* by using one of the standard query methods. We then know that *R* ∈ (*h*_*i*_*h*_*j*_)*F*_*H*\SO(3)/*H*_*h*_*j*_^T^, and so (42) is solved.

We note that as is the case in Section 6 (cf. (39)), the shifts of the fundamental domains *F*_*H*\SO(3)/*K*_ and *F*_*H*\SO(3)/*H*_ above all have the same volume, which is an important advantage of the double-coset approach presented in this paper.

### 7.2. Planar-Motion Alphabets Based on Wallpaper Groups

**Figure 5:**
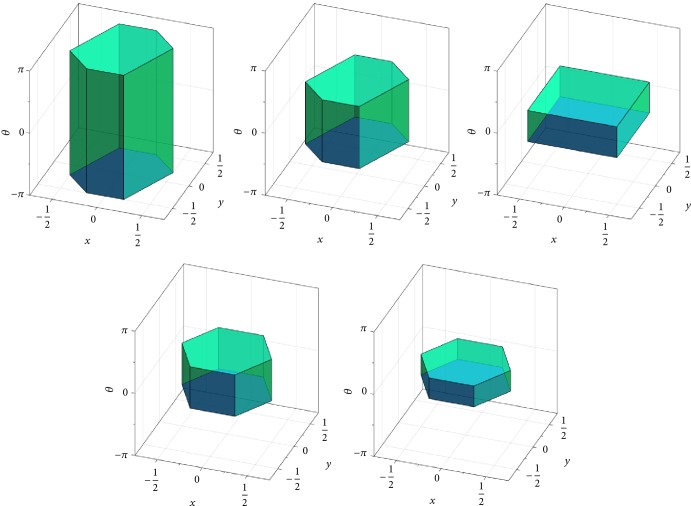
Center Voronoi cell *F*_p_*i*_\SE(2)_ for certain instances of the wallpaper groups (left to right, top to bottom) p_1_, p_2_, p_4_, p_3_, and p_6_.

As mentioned in Section 5, crystallographic groups are called wallpaper groups in the two-dimensional setting.  Figure 5 shows fundamental domains *F*_p_*i*_\SE(2)_ constructed using (19) for instances of the well-known wallpaper groups (cf. [69, Chap. 6]) p_1_, p_2_, p_4_, p_3_, and p_6_ (see also [55]), all of which are symmorphic. Here SE(2) is identified with *ℝ*^2^ × (−*π*, *π*) ⊂ *ℝ*^3^, with the *x* and *y* axes representing translations in *x* and *y* direction and the *z* axis representing the rotation angle *θ*. These fundamental domains are generated using the Euclidean metric ‖·−·‖_2_ on *ℝ*^3^, adapted so as to take into account the 2*π*-periodicity in the rotation angle *θ*. It is important to note that this metric is left- but not right-invariant (there is no bi-invariant metric on SE(2)). Therefore, the fundamental domains shown in Figure 5 are actually Voronoi rather than Voronoi-like cells (as is the case for SO(3), cf. (28)).

The group p_1_ consists solely of translations, constituting a parallelogrammatic lattice in the translational *x*-*y* plane. This results in a box with (irregular) hexagonal shape in the *x*-*y* plane and height 2*π* as the fundamental domain *F*_p_1_\SE(2)_. In addition to the translations in p_1_, the wallpaper group p_2_ also contains a rotation of order two (*i.e.*, with angle *π*). Therefore, the fundamental domain *F*_p_2_\SE(2)_ also has a hexagonal shape in the translational plane, but the height is only *π* (from −*π*/2 to *π*/2) instead of 2*π*. The group p_4_ is a group with rotations of order four (*i.e.*, with angles *π*/2, *π*, and 3*π*/2), as well as translations in a square lattice. Thus the fundamental domain *F*_p_4_\SE(2)_ has the shape of a square in the translational plane, with its height being *π*/2 (from −*π*/4 to *π*/4). The groups p_3_ and p_6_ both have a hexagonal translation lattice. In addition to these translations, p_3_ contains rotations of order three (rotation angles 2*π*/3 and 4*π*/3), while p_6_ contains rotations of order six (rotation angles *π*/3, 2*π*/3, *π*, 4*π*/3, and 5*π*/3). Both fundamental domains *F*_p_3_\SE(2)_ and *F*_p_6_\SE(2)_ have a regular hexagonal shape in the translational plane, with a height of 2*π*/3 (from −*π*/3 to *π*/3) and *π*/3 (from −*π*/6 to *π*/6), respectively.

**Figure 6:**
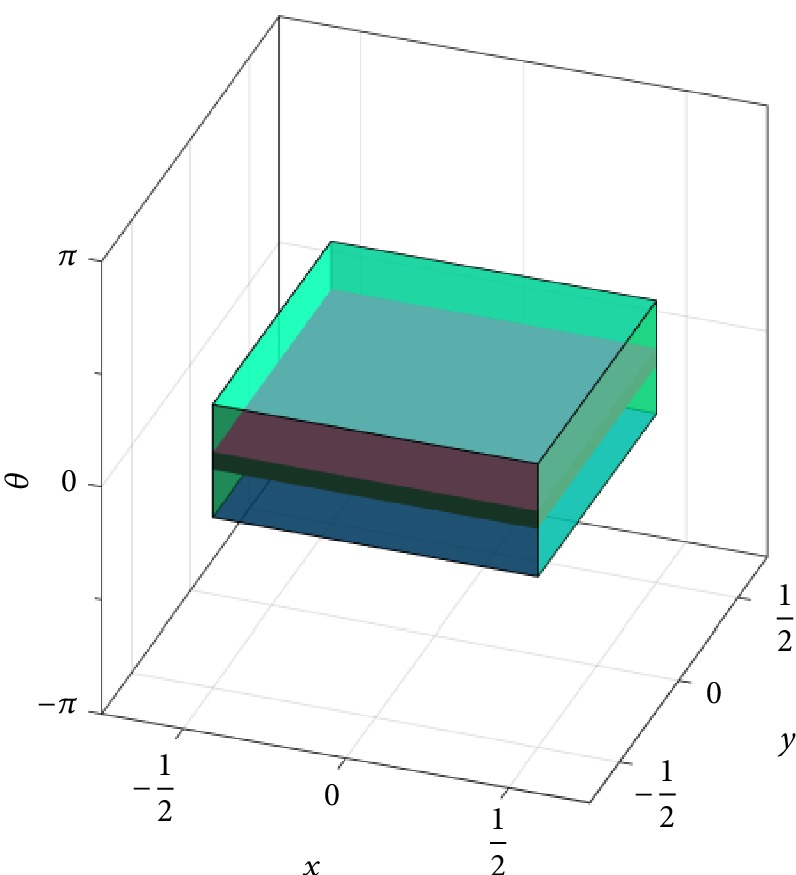
Center Voronoi cell *F*_Γ\SE(2)_ in single-coset space (emerald region) based on an instance of the wallpaper group Γ = p_4_, and center Voronoi cell *F*_Γ\SE(2)/Δ_ in double-coset space (ruby region) with Δ = C_5_⋉{0} as the group of rotations of order five.

As an example of a planar-motion alphabet similar to the one described in Section 6, let us consider the double-coset space Γ\SE(2)/Δ with Γ≔p_4_ and Δ≔C_2*n*−1_⋉{0} < SE(2), where

(45) $$\begin{array}{c} C_{2n-1}≔\left\{\left[\begin{array}{cc} cos\;\theta_{j} & -sin\;\theta_{j}\\ sin\;\theta_{j} & cos\;\theta_{j} \end{array}\right]:\theta_{j}=\frac{2\pi j}{2n-1},\;\;j=0,\ldots ,2\left(n-1\right)\right\}<SO\left(2\right) \end{array}$$

is the group of rotations of order 2*n* − 1 (*n* ∈ *ℕ*). It is Γ∩Δ = {*e*} and so we can construct a fundamental domain *F*_Γ\SE(2)/Δ_ as a Voronoi-like cell using (20). This is shown in Figure 6 for *n* = 3. In fact, because the left-invariant metric used here is also invariant under purely rotational actions from the right, the fundamental domain *F*_Γ\SE(2)/Δ_ is a classical Voronoi cell here, too (again as in the case of SO(3), cf. (29)). Analogously as in Section 6, we can use the alphabet Γ × Δ < SE(2)^2^ for an equivolumetric quantization of SE(2), which at the same time allows for a very uniform sampling of the group. Because the scaling of the translational lattice in the wallpaper groups is arbitrary, we can make the alphabet Γ × Δ arbitrarily fine by reducing the translational scaling in p_4_ and increasing the parameter *n* above.

To illustrate the use of the planar-motion alphabets constructed above, let us consider the SE(2) trajectory *g*(*τ*)≔(*R*(*τ*), **t**(*τ*)), *τ* ∈ [0,2*π*), where *R*(*τ*) is a rotation by an angle of *τ* and

(46) $$\begin{array}{c} t\left(\tau \right)≔{\left[4\;cos\;\tau ,6\left(\frac{\tau }{2\pi }\right)-3\right]}^{T}. \end{array}$$

We may discretize *g* at the five equidistant time points *π*(1 + 2*k*/5), *k* = −2,…, 2. As a motion alphabet for SE(2), let us use Γ × Δ = p_4_ × C_5_. We can denote the elements of C_5_ by *δ*_*j*_ with the index *j* as in (45). Let us denote the elements of p_4_ as *γ*_*lmn*_, where *m* ∈ *ℤ* and *n* ∈ *ℤ* denote the translation in *x* and *y* direction, respectively, while *l* ∈ {0,1, 2,3} indicates a rotation by an angle of *lπ*/2. The continuous motion trajectory *g* can now be expressed as the sentence (*γ*_2,3,−2_, *δ*_3_), (*γ*_2,−1,−1_, *δ*_4_), (*γ*_2,−4,0_, *δ*_0_), (*γ*_2,−1,1_, *δ*_1_), (*γ*_2,3,2_, *δ*_2_).

**Figure 7:**
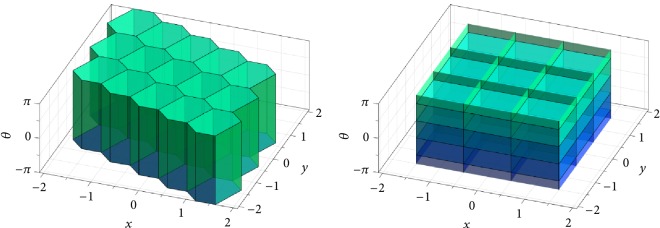
Tessellation of SE(2) illustrated in *ℝ*^2^ × (−*π*, *π*), based on the fundamental domain (left) *F*_p_1_\SE(2)_ and (right) *F*_p_4_\SE(2)_ (conceptual plot).

To close this section, we shall discuss how such decoding problem can be solved efficiently. We can use the same coarse-to-fine search scheme that we used in the purely rotational case in the previous section: In a first step, for a given element *g* ∈ SE(2), we find *γ* ∈ p_4_ such that *g* ∈ *γF*_Γ\SE(2)_. This step is particularly easy in the case of p_4_ when compared with,* e.g.*, the group p_1_ (with anisotropic translational lattice). In fact, as implied by Figure 7, in the case of p_4_ the above step can be realized by appropriately rounding off (in the decimal sense) the translational components of *g*, as well as the rotation angle. In the case of p_1_, on the other hand, we would have to compute the distances of *g* to the Voronoi centers. In a second step, we search for the shifted fundamental domain *γ*′*F*_Γ\SE(2)/Δ_*δ* containing the pulled-back element *γ*^−1^*g* by a purely rotational search. The decomposition of *g* then reads (*γγ*′)*Qδ* with *Q* = (*γγ*′)^−1^*gδ*^−1^ ∈ *F*_Γ\SE(2)/Δ_. Of course it is also possible to first treat the translational part of *g*and then, say, the rotational part. With appropriate modifications, the above coarse-to-fine decoding scheme can also be used with the fine alphabet for SE(3) developed in Section 6.

## 8. Comparisons and Applications

To clearly demonstrate the advantageous potential of our proposed discretization and decoding algorithms, we provide comparisons of performance with existing methods and introduce a hybrid sampling method to take advantage of the speed and low dispersion properties.

### 8.1. The Accuracy and Speed of Rounding Off Motions

#### 8.1.1. Uniformity of Sampling on SO(3)

Discretization on SO(3) is an important application of this work, which gives equivolumetric decomposition of the group in the sense that any rotation is located inside an identical Voronoi cell. The uniformity is essential to evaluate how good a sampling method is, and can be measured by* dispersion*,* discrepancy*,* consistency*, etc., as introduced in Section 2.2. For the computations of dispersion and consistency in this work, *ρ*(*R*, *R*_*s*_)≔‖log^∨^(*R*^*T*^*R*_*S*_)‖_2_ is the distance metric defined in Section 3.2.

We compare the dispersion (3), consistency (4), and uniformity (7) of our method in sampling from SO(3) with uniform Euler angles and uniform sampling using Hopf fibration [53]. For the dispersion comparison, we randomly generate 10000 rotations and compute the distance with the nearest sample. The maximum value of these 10000 resulting distances approximates the dispersion. For the consistency, on the other hand, for each sample, we compute the distance to its nearest sample and take the standard deviation. And for the uniformity measure, we compute the number of nearest neighbors of each sample.

For our method, we decompose SO(3) using the double-coset space *H*\SO(3)/*K*, where *H* is the group of rotational symmetries of the icosahedron and *K* = *gHg*^−1^, where *g* is chosen such that

(47) $$\begin{array}{c} {log}^{\vee }g≔{\left[0.4359,-0.07692,-0.1282\right]}^{T}. \end{array}$$

We found that the 181 shifted fundamental domains *h*_*i*_*F*_*H*\SO(3)/*K*_*k*_*j*_ with center *h*_*i*_*k*_*j*_ closest to the identity are sufficient to cover *F*_*H*\SO(3)_. And for the Euler angle, we choose *ZYZ* parameterization and uniformly generate *α* and *γ* within their range [−*π*, *π*] and *β* such that cos *β* ∈ [−1,1].

In terms of the dispersion, our method is higher than the other two methods: the dispersion of ours is 0.4291, Hopf fibration method is 0.2690, and Euler angle method is 0.3401. However, for the consistency, ours can achieve 0 deviation, while Hopf is 0.0264 and Euler angle is 0.0865. This consistency result is a significant advantage of our method in the sense that the sampling grid always has equi-distance edges. Also since the number of nearest neighbors with minimum distance for our method is always 2, the uniformity is 2. This also outperforms the other two methods, where Hopf fibration has a uniformity of 0.25, and Euler angle is only 0.0303. The results show that our method can be used in uniformly sampling rotations from SO(3).

#### 8.1.2. Computational Time of SO(3) Nearest Neighbor Search

Another key factor for performance evaluation is the running time when searching for the nearest sample for a random rotation. A common and efficient way is using the Euler angle parameterization. Suppose we are given a set of sample points constructed using Euler angles (either with or without cos^−1^ sampling, or Lattman's diagonalization of the metric tensor, as discussed in the Introduction). Then given an arbitrary rotation, *R*, one can compute its Euler angles (*α*_*R*_, *β*_*R*_, *γ*_*R*_) and attempt to round off to the nearest Euler angles in the sample set as ([*α*_*R*_], [*β*_*R*_], [*γ*_*R*_]). This is simple and fast to compute, but because Euler angles are not an equi-metric spacing, the resulting rounded rotation matrix [*R*] = *R*_3_([*α*_*R*_])*R*_1_([*β*_*R*_])*R*_3_([*γ*_*R*_]) may not be the closest of the sampled rotations to *R*. In contrast, our method is both fast and accurate in the sense of metric round-off.

To illustrate this, we perform a comparison here at an Intel Core i7-4790 CPU at 3.60  GHz × 8 and with Matlab R2018b. For our method, we use the double-coset space *H*\SO(3)/*H*, where we have a faster version of the decoding algorithm. Comparisons are performed with the Euler angle searching method (described above). 1000 random rotations are generated and localized to the nearest sample from the sampling list. The accuracy of computing the nearest neighbor is evaluated using the brute-force nearest neighbor search (*i.e.*, minimization of the distance *ρ*(*R*, *h*_*i*_*h*_*j*_) with respect to both *i* and *j*).

**Figure 8:**
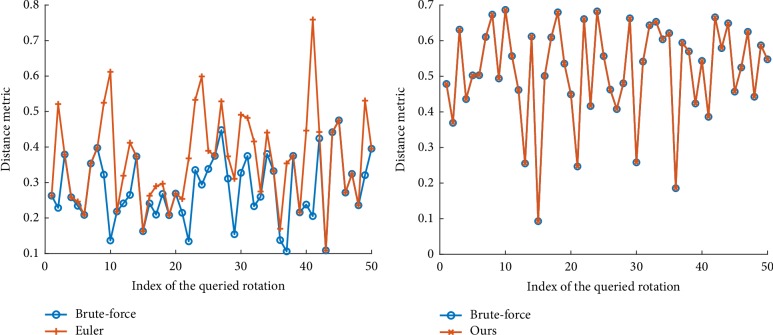
Comparisons of the minimum distance between the queried rotations to the set of samples. The true values are computed using the brute-force nearest neighbor search, which is shown in blue curve. The Euler angle search (left figure) sometimes returns higher values of distance, but ours (right figure) can always give the correct answer.

The result shows that the proposed decoding algorithm yields an average runtime for each search at around 55.9*μs* in our method, while the Euler angle method runs 53.6*μs* per rotation. Both methods are at the same level of efficiency, but ours outperforms in terms of round-off accuracy.  Figure 8 shows the minimum distance between the queried rotations (50 of all the testing rotations are shown for a clearer plot) to the set of samples. Ours can always find the true nearest neighbor, while Euler angle sometimes returns the sample which is not the nearest to the queried rotation.

### 8.2. Combining the Benefits of Speed and Good Dispersion Properties

Sampling methods such as those based on the Hopf fibration were designed for good performance in terms of minimal dispersion and consequently outperform both Euler angles and our sampling scheme in terms of minimizing dispersion. Ours was designed for rapid query. However, if one wants both, it is possible to simply combine them in a natural way as follows:

(1) Partition any given set of sample rotations with desirable properties (such as dispersion and discrepancy) by determining in which shard each sample point belongs.

(2) Given an arbitrary rotation, determine to which double-coset fundamental domain it belongs.

(3) Compute the distance between the arbitrary given rotation and all sample points in the same shard, and those in the nearest surrounding shards.

In this scheme the number of sample points could be in the same order or even much higher than the number of shards.

We perform a numerical experiment to verify this proposed hybrid searching method using the *H*\SO(3)/*H* decomposition. As a preprocessing step, we first compute the nearest neighbors for each of the 60 elements in *H*, and we find that each rotation is surrounded by 12 neighbors. This step is to construct a connectivity map for the single-coset space or, in other words, for each icosahedron cell. Then we uniformly sample 10000 rotations using Hopf fibration, which is known to have low dispersion and discrepancy. For each sample, we decompose the rotation group via our proposed fast decoding algorithm and precompute a list of index pair, which locates the cell and shard of those samples. Afterwards, we randomly generate 1000 rotations and the goal is to find their closest sampling rotations. For each of the random rotations, we decompose it, locate the cell (determined by the first index), and then calculate the minimum distance with all the samples located in the same cell and the 12 neighboring cells. By using this hybrid method, the running time is around 5 times faster than the brute-force searching method, and the resulting minimum distance is verified to be 100% correct.

Another experiment has also been performed using the *H*\SO(3)/*K* decomposition. The difference with the previous test is the preprocess, where here we precompute the nearest neighbors for the 3600 elements, *i*.*e*., *h*_*i*_*k*_*j*_ ∈ *H* × *K* where *i*, *j* = 1,2,…, 60. This is equivalent to finding the neighbors of each shard in the double-coset space. The same sampling and testing sets are input into the hybrid algorithm and we achieve a speedup of around 20 times than the brute-force method. Also, the resulting minimum distance is verified as 100% accurate.

## 9. Conclusion

Robot tasks involve continuous motions in space. The quantization of these motions by introducing a class of motion alphabets has been established in this paper. With such an alphabet, continuous motion trajectories can be captured with finite words/sentences. It was demonstrated in some examples how the possibility of constructing fundamental domains for coset and double-coset spaces as Voronoi or Voronoi-like cells can be used to solve this decoding or “signals to symbols” problem efficiently via a coarse-to-fine search scheme. The performance, such as uniformity, of the proposed group discretization method and the fast decoding algorithms are compared with other existing methods. The alphabets developed here will be used in the future to facilitate the connection between advances in artificial intelligence (such as the use of artificial neural networks) and physical robots acting in the world.
